# Sign inversion in magnetic circularly polarised luminescence of fused aromatics with 1.6 T N-up/S-up Faraday geometry†

**DOI:** 10.1039/d0ra09233a

**Published:** 2021-01-05

**Authors:** Hayato Toda, Nobuyuki Hara, Michiya Fujiki, Yoshitane Imai

**Affiliations:** Department of Applied Chemistry, Faculty of Science and Engineering, Kindai University 3-4-1 Kowakae, Higashi-Osaka Osaka 577-8502 Japan y-imai@apch.kindai.ac.jp; Graduate School of Materials Science, Nara Institute of Science and Technology Takayama Ikoma Nara 630-0192 Japan

## Abstract

Although 12 diamagnetic fused aromatics with or without substituents exhibit mirror-symmetric magnetic circularly polarised luminescence (MCPL) through N-up and S-up Faraday geometries under a magnetic field intensity of 1.6 T, their signs (single and multiple) and magnitudes depend strongly on either the aromatic structures or the peripheral positions of the substituents.

## Abstract 2

Diamagnetic fused aromatics exhibit mirror-symmetric magnetic circularly polarised luminescence (MCPL) depend on either the aromatic structures or the peripheral positions of the substituents through N-up and S-up Faraday geometries under a 1.6 T magnetic field.

## Graphical abstract

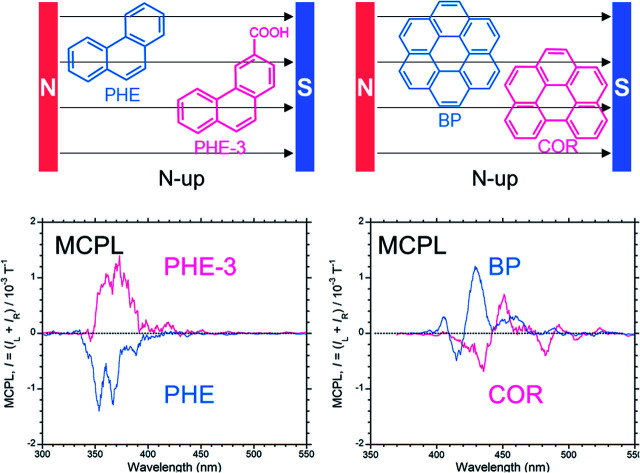

## Introduction

In recent years, highly controlled chirogenesis from organic, inorganic, and polymeric luminophores in the photoexcited state (ES) and the ground state (GS) has received considerable attention in photonic materials science and engineering.^1^ In particular, sophisticated organic luminophores that can emit circularly polarised luminescence (CPL) with a high dissymmetry ratio (*g*_CPL_), high quantum yield (*Φ*_F_), and additional functionality remain challenging to produce and are in great demand from the perspective of extensibility.^2^

Generally, to afford the ambidextrous (+ and −) signs of CPL signals, enantiomerically pure luminophores with twisted and/or chirally distorted configurations and conformations designed rationally in the ES and GS are imperative. This can be achieved by choosing rigid chiral π-conjugated frameworks associated with both (*S*)- and (*R*)-stereogenic centres and/or both *P*- and *M*-stereogenic bonds at their peripheral positions. Such enantiomeric pairs are not always available as the starting source materials, however, and therefore, enantiomeric CPL-functioned substances often require multiple-step synthesis.

Recently, we demonstrated that simple control of ambidextrous CPL signs using semi-rigid chiral luminophores with few rotor axes is possible if appropriate achiral solvents and chiral additives with few rotor axes are chosen.^3^

Contrarily, for affording such ambidextrous CPL in the ES, interaction between unpolarised light and external magnetic field,^4^ the so-called Faraday and polar-Kerr effects, is a versatile physical source, as is the use of electric field and vortex fluidic solvents. In fact, we showcased mirror-image magnetic circularly polarised luminescence (MCPL) from *D*_2h_-symmetrical planar pyrene and six-coordinate *C*_3_-symmetrical lanthanides with achiral tris(β-diketonate) complexes.^5^ Even *C*_2_-symmetrical binaphthyl bearing two achiral pyrene rings showed nearly mirror-symmetrical MCPL.^5^

Although ambidextrous MCPL spectra under 1.6 T are easy to achieve by choosing both N-up and S-up Faraday geometries,^5^ questions remain unanswered as to whether (i) the resulting MCPL sign among all diamagnetic aromatics is commonly monosignate, (ii) MCPL signs are determined solely by the N-up/S-up geometry, (iii) the MCPL sign is affected by the substituents at peripheral positions, and (iv) the MCPL magnitude is determined by the number of fused rings.

To address these queries, we experimentally investigated the spectral characteristics of MCPL and unpolarised photoluminescence (PL) with *H*_0_ = 1.6 T and those of CPL and PL with *H*_0_ = 0.0 T, including the dissymmetry ratio (*g*_MCPL_), spectral profiles, and *Φ*_F_ of several fused aromatic molecules dissolved in common organic solvents; some of the solvents were dispersed in a PMMA (poly(methyl methacrylate)) film by applying both the N-up and S-up geometries.

## Experimental

### Materials

PHE, NP, NP-1 and PMMA were purchased from FUJIFILM Wako Pure Chemical (Osaka, Japan). ANT-1 was purchased from Combi-Blocks (San Diego, USA). ANT, ANT-2, NP-2, BP and COR were purchased from Tokyo Chemical Industry (Tokyo, Japan). PHE-2 and PHE-3 were purchased from Merck-Sigma-Aldrich Japan (Tokyo, Japan). Spectroscopic grade solvents (CHCl_3_, THF, cyclohexane (CHX), and dimethyl sulfoxide (DMSO)) were purchased from Dojindo Laboratories (Kumamoto, Japan).

### Instrumentations

#### MCPL, CPL and PL measurements

MCPL, CPL and PL spectra were acquired using a JASCO (Hachioji-Tokyo, Japan) CPL-300 spectrofluoro-polarimeter equipped with and without a JASCO PM-491 permanent magnet (1.6 T) at room temperature at a forward scattering angle with 0° upon excitation by unpolarised monochromatic incident light with a 10 nm bandwidth. PMMA film samples were prepared by spin-coating PHE-containing PMMA CHCl_3_ solution (1.0 × 10^−2^ M) at 3000 rpm (Opticoat MS-A100, Mikasa, Tokyo, Japan). The excitation wavelengths were 355 nm for ANT-1 and ANT-2 in DMSO; 340 nm for ANT in DMSO; 280 nm for NP-1, NP-2, and NP in DMSO; and 270 nm for PHE in DMSO, CHCl_3_, THF, and the PMMA film, as well as for PHE-2 and PHE-3 in DMSO. A 5 mm and 10 mm pathlength cell were used for solution-state MCPL and CPL measurements, respectively. BP in DMSO was excited at 350 nm and COR in DMSO was excited at 340 nm.

#### UV-Vis absorption measurement

Solution UV-Vis absorption spectra of the luminophores were measured with a JASCO V-670KU spectrophotometer with 10 mm pathlength. The value of *Φ*_F_ was obtained with a quantum yield spectrometer (Hamamatsu Photonics, Hamamatsu, Shizuoka, Japan, model C9920-02).

## Results and discussion

**Fig. 1:**
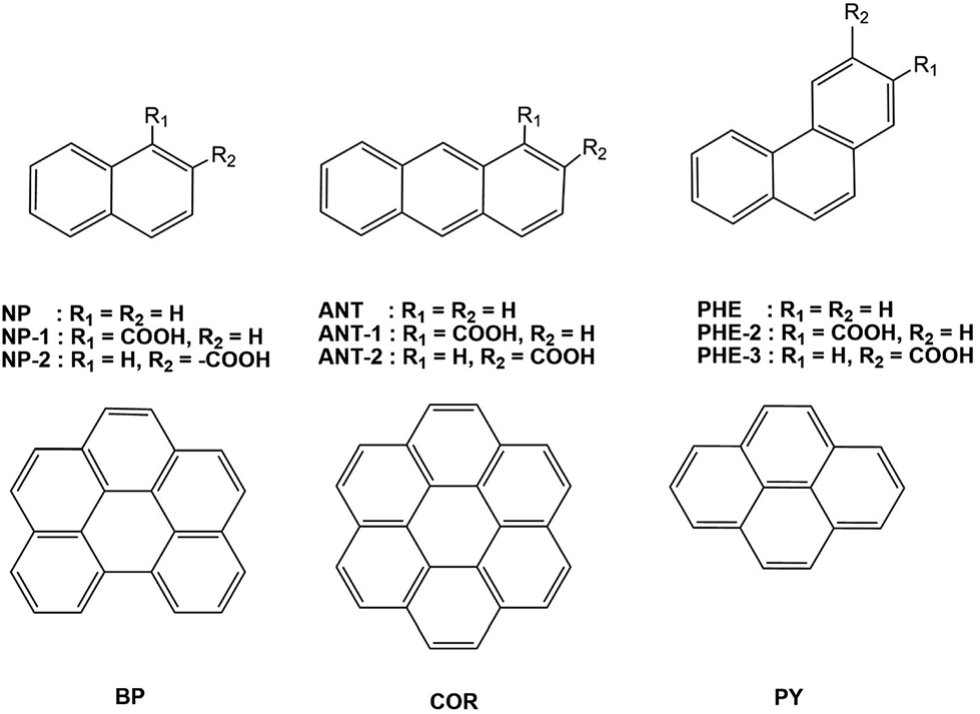
Chemical structures of naphthalene (NP), anthracene (ANT), phenanthrene (PHE), benzo[ghi]perylene (BP), coronene (COR), and pyrene (PY) without or with substituents at two different peripheral positions (NP-1, NP-2, ANT-1, ANT-2, PHE-2, and PHE-3).

We chose six unsubstituted aromatics: naphthalene (NP), anthracene (ANT), phenanthrene (PHE), benzo[ghi]perylene (BP), coronene (COR), and pyrene (PY).^5*a*^ To investigate substituent effects, we also studied six NP, PHE, and ANT derivatives carrying electron-withdrawing carboxylic acids at two different peripheral positions (NP-1 and NP-2, PHE-2 and PHE-3, and ANT-1 and ANT-2), as shown in Fig. 1.

We observed that the 12 fused aromatics with or without the substituents exhibit mirror-symmetric MCPL spectra under N-up and S-up Faraday geometries. Among these aromatics, BP and COR exhibit unexpectedly complex MCPL spectral profiles with multiple signs. The other aromatics have simple monosignate MCPL spectra, although their signs and magnitudes largely depend on either the topology of the aromatics or the position of the substituents. All UV-visible absorption spectra of the aromatic luminophores in solutions before the MCPL, CPL, and PL spectral measurements are depicted in Fig. S1–S5, ESI.†

**Fig. 2:**
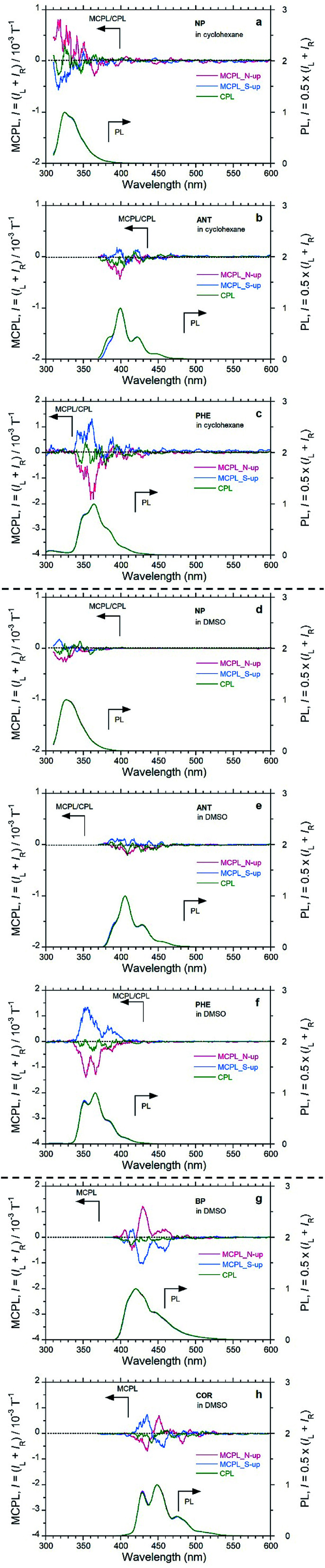
MCPL spectra at *H*_0_ = 1.6 T (red and blue lines denote N-up and S-up geometries, respectively), CPL spectra at *H*_0_ = 0.0 T (green line), and the corresponding PL spectra in dilute solutions (1.0 × 10^−3^ M); pathlengths were 5 mm for MCPL and 10 mm for CPL. (a) NP in CHX excited at 280 nm, (b) ANT in CHX excited at 340 nm, and (c) PHE in CHX excited at 270 nm, (d) NP in DMSO excited at 280 nm, (e) ANT in DMSO excited at 340 nm, and (f) PHE in DMSO excited at 270 nm. (g) BP in DMSO excited at 350 nm and (h) COR in DMSO excited at 340 nm.

Firstly, we compared the MCPL and PL spectra under 1.6 T with the N-up and S-up geometries associated with the corresponding CPL/PL spectra without the magnetic field (Fig. 2 and Table 1). When PL intensity is normalised so that PL = (*I*_L_ + *I*_R_)/2 = 1.0 from the raw PMT DC voltage, the MCPL and CPL spectra can be normalised by the common equations *g*_MCPL_ at 1.6 T = (*I*_L_ − *I*_R_)/[(*I*_L_ + *I*_R_)/2] and *g*_CPL_ at 0 T = (*I*_L_ − *I*_R_)/[(*I*_L_ + *I*_R_)/2], where *I*_L_ and *I*_R_ denote the intensities of the left- and right-MCPL and CPL components, respectively, upon excitation by unpolarised light. Secondly, the *g*_MCPL_ values at 1.6 T from an apparent MCPL spectrum were normalised to be *g*_MCPL_ per 1.6 T and expressed in units of T^−1^. This is because *g*_MCPL_ may depend on the strength of the magnetic field to some extent.

**Table 1:** MCPL characteristics (normalised at 1.6 T) of NP, ANT, PHE, BP, COR, and PY in common organic solvents (1.0 × 10^−3^ M) and PMMA film cast from a dilute CHCl_3_ solution

Entry	Medium	*Φ*_F_	*g*_MCPL_ (*λ*_MCPL_ in nm)/10^−3^ T^−1^	
			N-up	S-up
NP^a^^,^^b^	CHX	0.005	+0.77 (317)	−0.56 (316)
	DMSO	0.025	−0.26 (327)	+0.29 (318)
ANT^b^	CHX	0.196	−0.23 (399)	+0.07 (399)
	DMSO	0.198	−0.22 (409)	+0.12 (405)
PHE^a^	CHX^b^	0.033	−1.08 (361)	+0.85 (361)
	DMSO^a^	0.108	−1.06 (353)	+0.97 (353)
	THF^a^	0.048	−1.00 (349)	+0.86 (349)
	CHCl_3_^a^	0.032	−1.06 (347)	+1.76 (349)
	PMMA^a^^,^^c^	0.045	−1.63 (345)	+1.27 (345)
BP	DMSO	0.382	−0.27 (415)^a^	+0.17 (415)^a^
			+0.86 (429)^b^	−0.71 (428)^b^
COR	DMSO^a^	0.115	−0.58 (435)^a^	+0.68 (435)^a^
			+0.46 (451)^b^	−0.38 (458)^b^
PY^a^^,^^d^	CHCl_3_	n.d.	−8.2 (374)	+8.2 (374)
	PMMA	n.d.	−7.0 (374)	+7.0 (374)

a At 0–0′ band.

b At 0–1′ band.

c Fig. S6, ESI.

d Data taken from ref. 5*a*.

NP, ANT, and PHE in CHX emitted clear MCPL (Fig. 2(a–c)). Among these unsubstituted aromatics, PHE in CHX exhibited the highest |*g*_MCPL_| value, on the order of 10^−3^ T^−1^. The other two aromatics (NP and BP) in CHX have |*g*_MCPL_| values on the order of 10^−4^ T^−1^. In addition, these luminophores in DMSO also exhibited clear MCPL signals (Fig. 2(d–f)). In particular, MCPL from PHE in DMSO is as strong as MCPL from PHE in CHX. For PHE, solvent effects and external matrix effects were also examined. PHE emitted clear MCPL in THF and CHCl_3_ solutions and in PMMA film (Fig. S6†). Interestingly, cyclic π-conjugated BP and COR also exhibited strong MCPL in DMSO (Fig. 2(g and h)).

The sign of MCPL from PHE and ANT is clearly negative (red lines; upper panel in Fig. 2, S7† and Table 1), and NP appears to exhibit positive-sign signals for an N-up Faraday geometry; this indicates that the N → S magnetic field relative to the incident light is applied. Conversely, a negative sign (blue lines; upper panel in Fig. 2 and S7† and Table 1) occurs for the S-up geometry, as expected. Both the N- and S-up Faraday geometries enable the verification of nearly mirror-image MCPL signals from achiral aromatic luminophores. Notably, the MCPL signals from NP in CHX and DMSO are oppositely signed, although the reason for this is unknown. This shows that the MCPL signs can be controlled not only by the direction of magnetic field but also by the solvent.

NP, ANT, PHE, BP, and COR do not exhibit clear CPL signals (green lines; upper panel in Fig. 2), reflecting the achiral planarity in the ES as well as in the GS in the absence of the 1.6 T magnet. Thus, our MCPL instrument with a magneto-optical set-up and the CPL instrument with a chiro-optical set-up are guaranteed to detect MCPL and CPL signals precisely from achiral luminophores, regardless of the presence or absence of the 1.6 T magnetic field.

Among the planar aromatics, the *g*_MCPL_, MCPL extremum wavelength (*λ*_MCPL_), and *Φ*_F_ (at 0.0 T) of PHE depend to some extent on the nature of the fluidic solvents and solidified film used, although the MCPL signs remain unchanged. For example, the *g*_MCPL_ values of PHE in CHCl_3_ and in PMMA are +1.76 × 10^−3^ T^−1^ at 349 nm (S-up) and −1.63 × 10^−3^ T^−1^ at 345 nm (N-up), respectively, while the |*g*_MCPL_| values in the other conditions range from 0.9 × 10^−3^ T^−1^ to 1.3 × 10^−3^ T^−1^. DMSO exhibits the highest *Φ*_F_ value, 0.108, while the other liquids and solid media exhibit lower *Φ*_F_ values of 0.03–0.05. This uniqueness of DMSO may arise from a highly polar and *C*_2v_-symmetrical structure with two C–C rotor axes.

The shortest MCPL wavelengths (*λ*_MCPL_) of NP, ANT, PHE, BP, COR, and PY are 316–317 nm (not prominent due to the noise), 345–361 nm, 415 nm, 435 nm, and 374 nm, respectively, corresponding to the vibronic 0–0′ PL bands of the monomer species in the media. This knowledge facilitates tailoring of solely the *λ*_MCPL_, which can be performed by choosing various well-known and/or well-designed aromatic luminophores ranging from the UV-visible to the near infrared (NIR) regions.

Between ANT and PHE (which are three fused-ring aromatics), PHE exhibits clear MCPL but ANT does not. We assume that it is more advantageous for a bent or curved π-structure to emerge and boost MCPL compared with a linear π-structure. BP and COR are examples of bent and curved aromatics. Furthermore, among the six aromatics, PY has the greatest |*g*_MCPL_| value, 7 × 10^−3^ to 8 × 10^−3^ T^−1^, which is exceptionally high compared with the other five values. PY is regarded as an extended PHE derivative with a two-fold symmetry. Furthermore, BP is considered as a PHE derivative fused with biphenyl moiety, while COR is considered a six-fold symmetrical structure composing two PHE blocks.

Contrary to expectations, we observed unique MCPL spectra with multiple signs for BP and COR in DMSO (Fig. 2(g and h)): a (+/−/+/+)-quadruple-sign sequence for BP with the N-up geometry and *vice versa* with the S-up geometry; a (−/+/−)-triple-sign sequence for COR with the N-up geometry, and *vice versa* with the S-up geometry. The apparent MCPL sign inversion characteristics appear to occur at the 0–0′, 0–1′, 0–2′, and 0–3′ vibronic transitions. A clear explanation for the origin of the multiple sign inversion could not be found. Our experimental results indicate that MCPLs induced by an external magnetic field are related to the photoexcited vibronic transition states arising from distorted C=C bonds in the fused ring and in-plane/out-of-plane C–H bending modes.

**Fig. 3:**
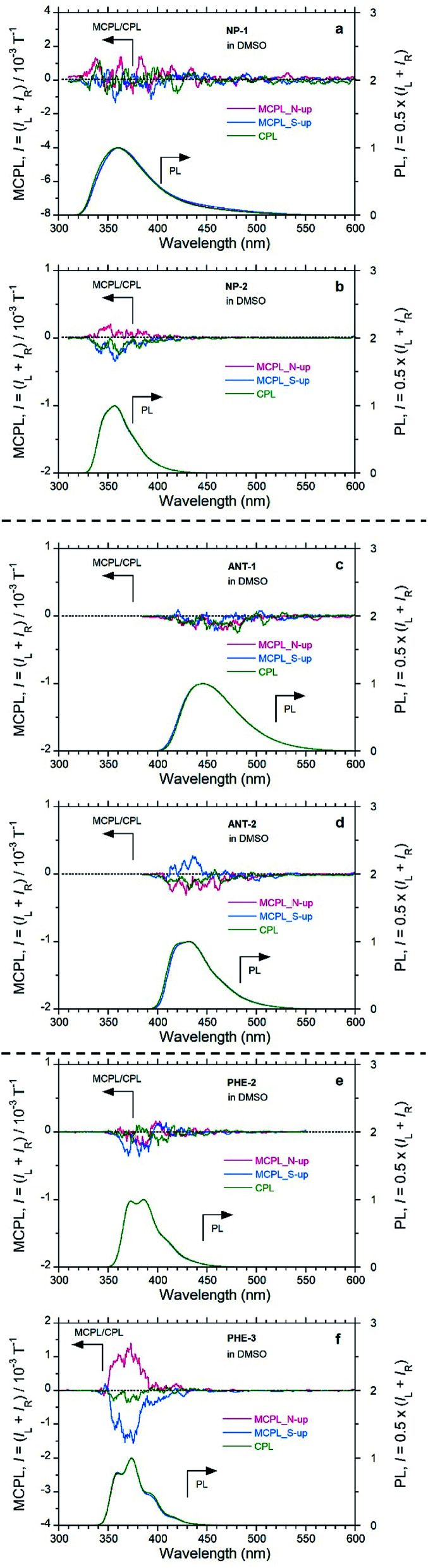
MCPL with *H*_0_ = 1.6 T (red and blue lines at N-up and S-up geometry, respectively), CPL (green line), and the corresponding PL spectra in dilute solutions (1.0 × 10^−3^ M; pathlengths: 5 mm for MCPL and 10 mm for CPL): (a) NP-1 and (b) NP-2 excited at 340 nm, (c) ANT-1 and (d) ANT-2 excited at 355 nm, and (e) PHE-2 and (f) PHE-3 excited at 270 nm.

The MCPL sign is significantly affected by the peripheral positions of the substituents (Fig. 3 and S8,† and Table 2). In the PHE derivatives in DMSO with the N-up geometry, PHE and PHE-2 exhibit (−)-sign MCPL, while PHE-3 exhibits (+)-sign MCPL. In the NP derivatives in DMSO with the N-up geometry, NP exhibits (−)-sign MCPL, while NP-1 and NP-2 exhibit commonly (+)-sign MCPL. In the ANT derivatives in DMSO with the N-up geometry, ANT, ANT-1, and ANT-2 exhibit the common (−)-sign MCPL. In addition to the dependence of MCPL sign inversion on substituents at peripheral positions, there is a trend in MCPL sign among PY, PHE and NP, which have successively fewer fused rings.

**Table 2:** MCPL characteristics (normalised at 1.6 T) of NP-1, NP-2, ANT-1, ANT-2, PHE-2, and PHE-3 in DMSO (1.0 × 10^−3^ M)

Entry	Medium	*Φ*_F_	*g*_MCPL_ (*λ*_MCPL_ in nm)/10^−3^ T^−1^	
			N-up	S-up
NP-1	DMSO	0.014	(+)^b^	(−)^b^
NP-2	DMSO	0.209	+0.12 (352)^b^	−0.20 (355)^b^
ANT-1	DMSO	0.690	(−)^b^	(−)^b^
ANT-2	DMSO	0.609	−0.14 (429)^b^	+0.13 (442)^b^
PHE-2	DMSO	0.312	(−)^b^	(−)^b^
PHE-3	DMSO^a^	0.150	+0.83 (374)	−0.88 (375)

a At 0–1′ band.

b Very weak signals although the MCPL sign is recognisable.

Regarding the |*g*_MCPL_| values, among PHE-3, NP-2, and ANT-2, the introduction of carboxylic acid groups slightly suppressed the |*g*_MCPL_| values by several times compared with the corresponding values for PHE, NP, and ANT (Fig. 3 and S8,† and Table 2). For the substituted derivatives, the MCPL signals were dramatically changed according to the substituent positions (Fig. 3 and Table 2). Thus, by introducing substituents at the proper peripheral positions, it was possible to suppress MCPL signals of the fused aromatics.

## Conclusions

We showcased ambidextrous chirogenesis from representative planar fused aromatic luminophores (NP, ANT, PHE, BP, and COR) and their derivatives with substituents, with the N-up and S-up Faraday geometries in a commercial CPL/PL spectrometer. The MCPL sign depended on the nature of the fused aromatics. Furthermore, although NP, ANT, and PHE exhibited mono-signate MCPL profiles with (−) or (+) signs, BP and COR exhibited complex MCPL with multiple (+)/(−)-sign sequences. Knowledge of the relation between MCPL characteristics and the properties of fused aromatics with or without substituents should aid in the rational design of more efficient MCPL luminophores in the UV-Vis-NIR region in the future, as solution states, in the solids, and in the solid film. The external magnetic field-induced ambidextrous mirror-symmetrical MCPL from achiral planar aromatics without any stereogenic centres and/or bonds in the ES is an alternative, versatile chirogenesis approach, as is the conventional chirogenesis approach using CPL substances containing stereogenic centres and/or bonds.

## Conflicts of interest

There are no conflicts to declare.

## Supplementary Material

RA-011-D0RA09233A-s001
