# Supplementary material for: Sign inversion in magnetic circularly polarised luminescence of fused aromatics with 1.6 T N-up/S-up Faraday geometry
Source: RSC Adv. 2021 Jan 5;11(3):1581–5. doi: 10.1039/d0ra09233a (PMC8693759; doi:10.1039/d0ra09233a)
Supplement: RA-011-D0RA09233A-s001 [file RA-011-D0RA09233A-s001.pdf]

Electronic Supplementary Material (ESI) for RSC Advances.
This journal is © The Royal Society of Chemistry 2020

Electronic Supporting Information for

Sign Inversion in Magnetic Circularly Polarised Luminescence of Fused Aromatics with 1.6 T N-up/S-up Faraday Geometry

Hayato Toda [a], Nobuyuki Hara [a], Michiya Fujiki [b] and Yoshitane Imai [a]*

[a] Department of Applied Chemistry, Faculty of Science and Engineering, Kindai University, 3-4-1 Kowakae, Higashi-Osaka, Osaka 577-8502, Japan. E-mail: y-imai@apch.kindai.ac.jp

[b] Graduate School of Materials Science, Nara Institute of Science and Technology, Takayama, Ikoma, Nara 630-0192, Japan

1. UV-vis absorption spectra of NP, NP-1, NP-2, PHE, PHE-2, PHE-3, ANT, ANT-1, ANT-2, COR, and BP.

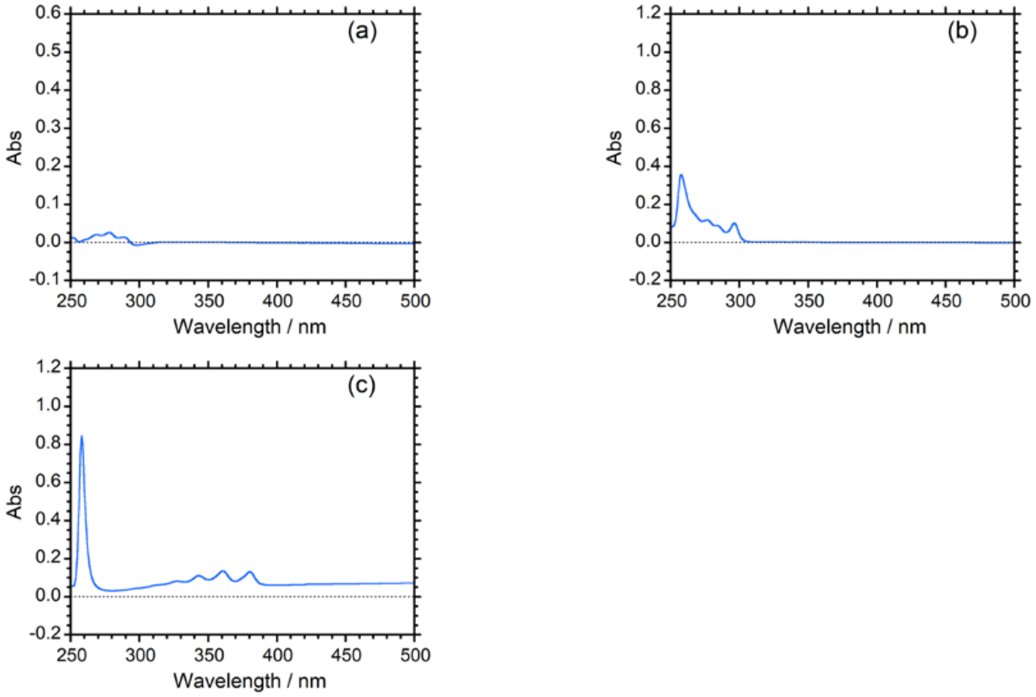

Fig. S1. UV-Vis spectra of (a) **NP** in DMSO (1.0 x 10^{-5} M), (b) **PHE** in DMSO (1.0 x 10^{-5} M) and (c) **ANT** in DMSO (1.0 x 10^{-5} M).

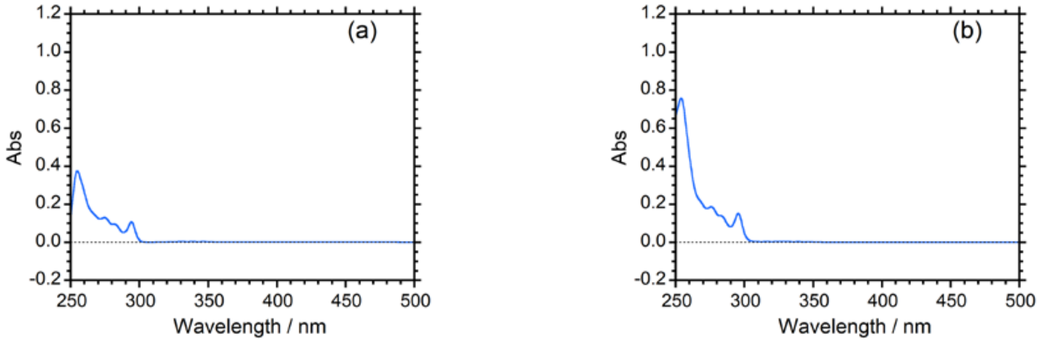

Fig. S2. UV-Vis spectra of (a) **PHE** in THF (1.0 x 10^{-5} M) and (b) **PHE** in $CHCl_3$ (1.0 x 10^{-5} M).

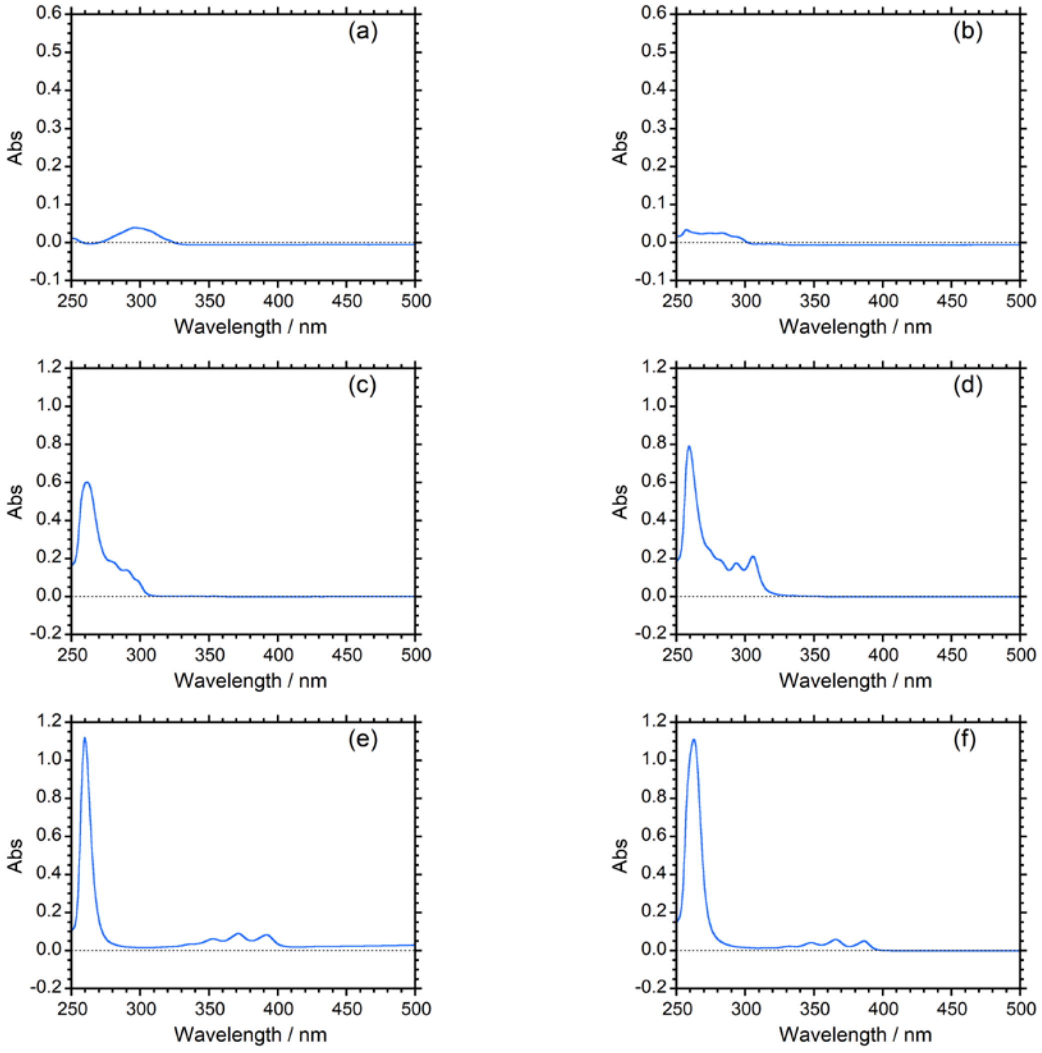

Fig. S3. UV-Vis spectra of (a) **NP-1** in DMSO (1.0 x 10^{-5} M), (b) **NP-2** in DMSO (1.0 x 10^{-5} M), (c) **PHE-2** in DMSO (1.0 x 10^{-5} M), (d) **PHE-3** in DMSO (1.0 x 10^{-5} M), (e) **ANT-1** in DMSO (1.0 x 10^{-5} M) and (f) **ANT-2** in DMSO (1.0 x 10^{-5} M).

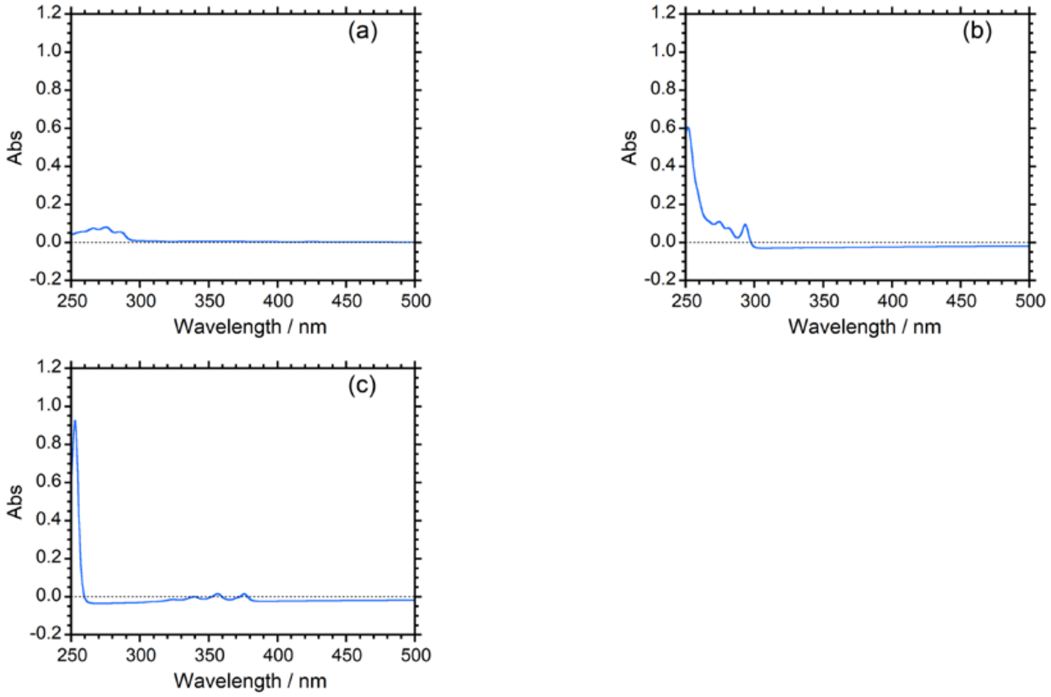

Fig. S4. UV-Vis spectra of (a) **NP** in CHX (1.0 x 10^{-5} M), (b) **PHE** in CHX (1.0 x 10^{-5} M) and (c) **ANT** in CHX (1.0 x 10^{-5} M)

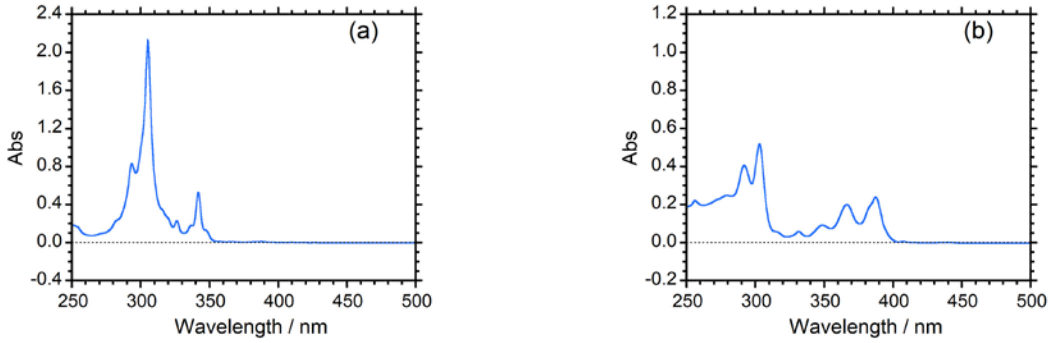

Fig. S5. UV-Vis spectra of (a) **COR** in DMSO (1.0 x 10^{-5} M) and (b) **BP** in DMSO (1.0 x 10^{-5} M).

2. MCPL, CPL and PL spectra of PHE in THF, $CHCl_3$, and PMMA

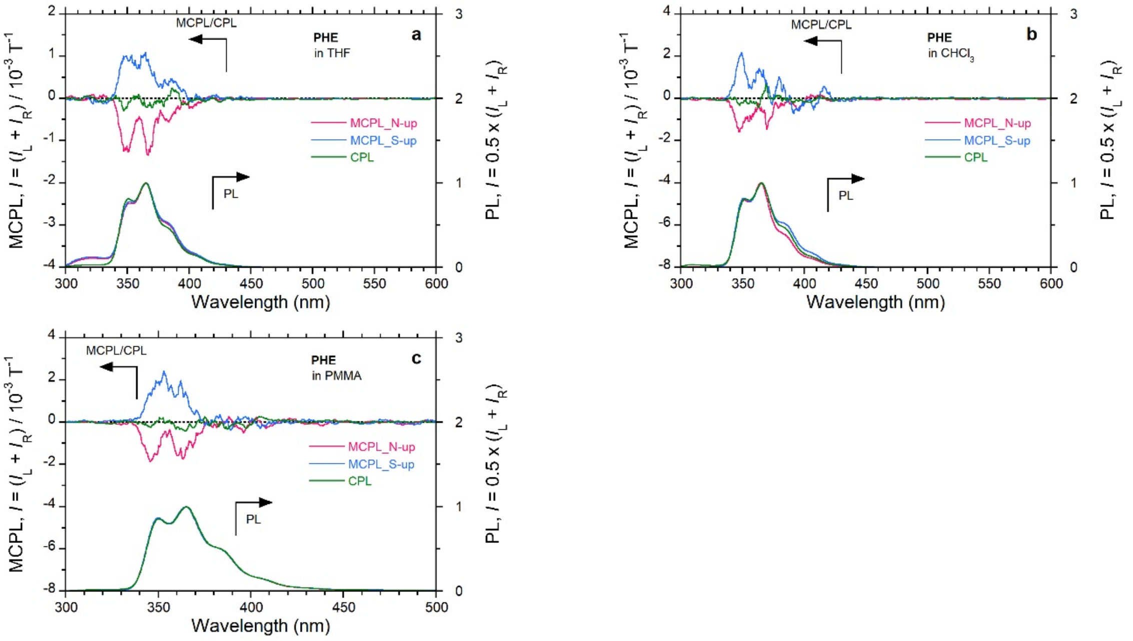

Fig. S6. MCPL and PL spectra of **PHE** in (a) THF, (b) $CHCl_3$, and (c) PMMA excited at 270 nm at H_0 = 1.6 T (red and blue lines at N-up and S-up geometry, respectively, associated with CPL spectra at H_0 = 0.0 T (green line) and the corresponding PL spectra.

3. G values and PL spectra of NP, NP-2, ANT, ANT-2, PHE, PHE-3, COR, and BP

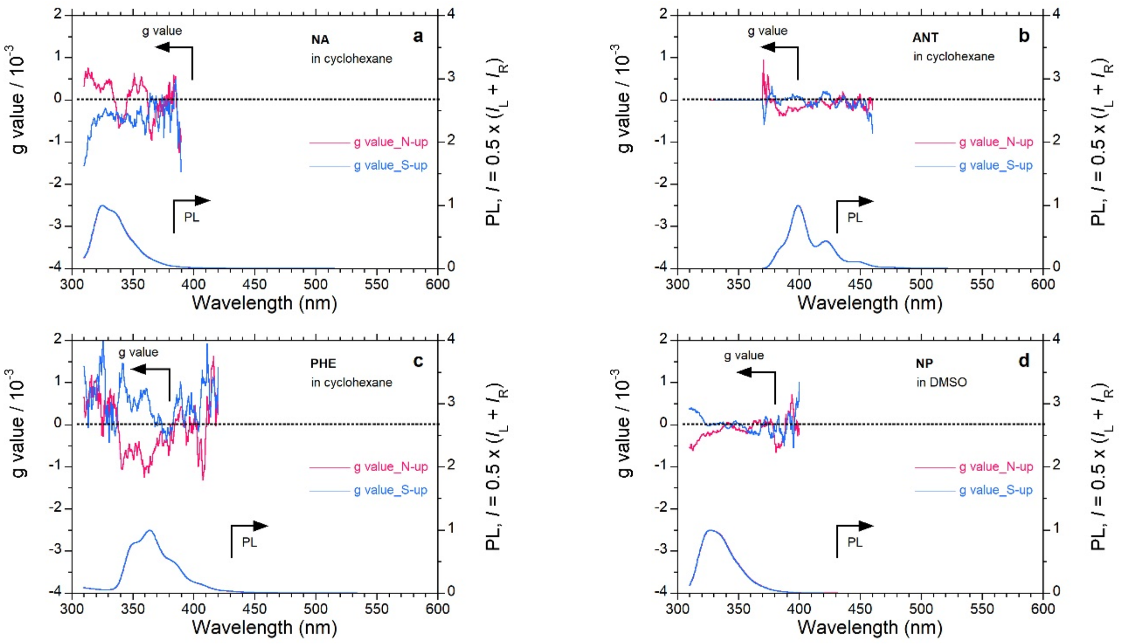

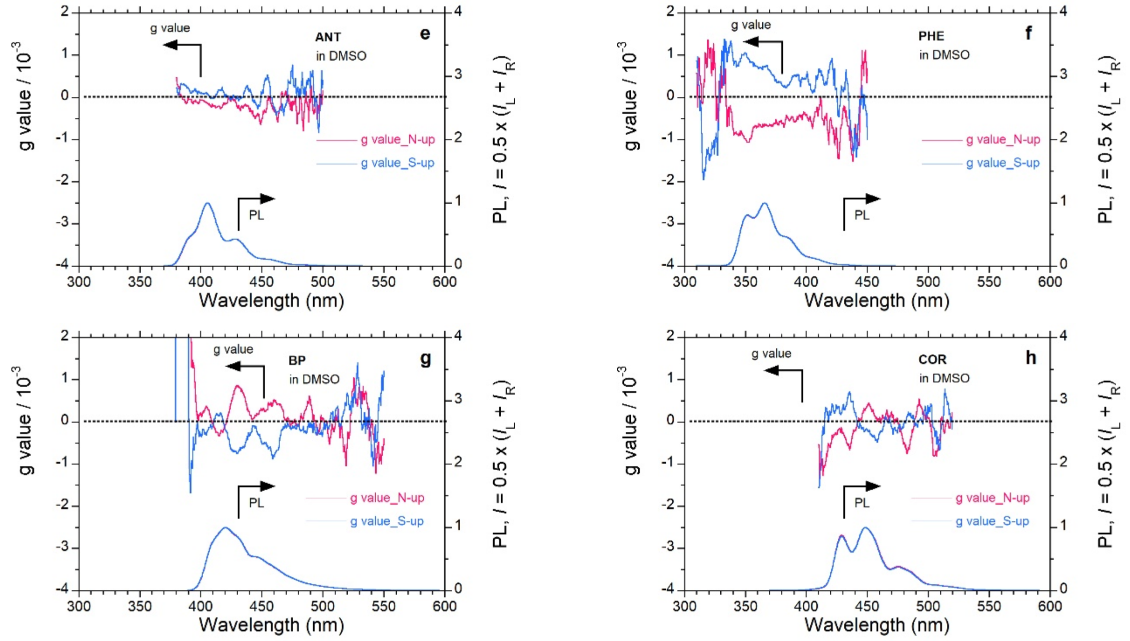

Fig. S7. G values and PL spectra of (a) **NP** in CHX, (b) **ANT** in CHX, (c) **PHE** in CHX, (d) **NP** in DMSO, (e) **ANT** in DMSO, (f) **PHE** in DMSO, (g) **BP** in DMSO and (h) **COR** in DMSO.

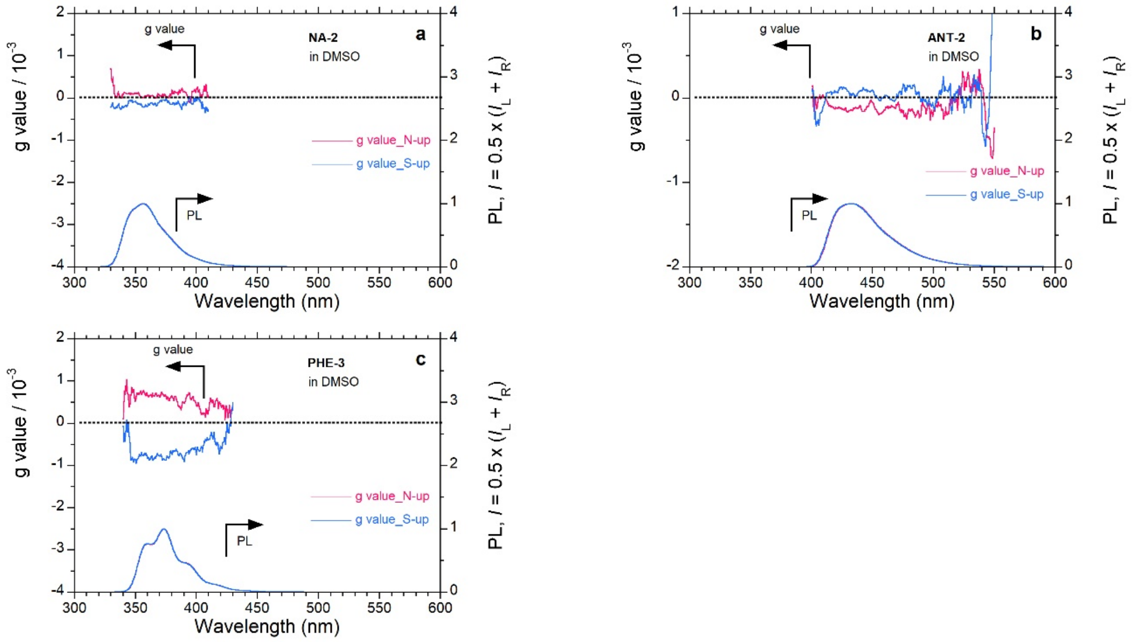

Fig. S8. G values and PL spectra of (a) **NP-2** in DMSO, (b) **ANT-2** in DMSO and (c) **PHE-3** in DMSO.
